# Integrating molecular pathway with genome-wide association data for causality identification in breast cancer

**DOI:** 10.1007/s12672-024-01125-7

**Published:** 2024-07-02

**Authors:** Yan-Shuang Li, Hong-Chuan Jiang

**Affiliations:** Department of Breast Surgery, Beijing Chaoyang Hospital, Capital Medical University, Beijing, 100020 China

**Keywords:** Breast cancer, Pyruvate metabolism, Pathway genes, Targeted drugs, Mendelian randomization

## Abstract

**Objective:**

The study purpose was to explore the causal association between pyruvate metabolism and breast cancer (BC), as well as the molecular role of key metabolic genes, by using bioinformatics and Mendelian randomization (MR) analysis.

**Methods:**

We retrieved and examined diverse datasets from the GEO database to ascertain differentially acting genes (DAGs) in BC via differential expression analysis. Following this, we performed functional and pathway enrichment analyses to ascertain noteworthy molecular functions and metabolic pathways in BC. Employing MR analysis, we established a causal association between pyruvate metabolism and the susceptibility to BC. Additionally, utilizing the DGIdb database, we identified potential targeted medications that act on genes implicated in the pyruvate metabolic pathway and formulated a competing endogenous RNA (ceRNA) regulatory network in BC.

**Results:**

We collected the datasets GSE54002, GSE70947, and GSE22820, and identified a total of 1127 DEGs between the BC and NC groups. GO and KEGG enrichment analysis showed that the molecular functions of these DEGs mainly included mitotic nuclear division, extracellular matrix, signaling receptor activator activity, etc. Metabolic pathways were mainly concentrated in PI3K−Akt signaling pathway, Cytokine−cytokine receptor binding and Pyruvate, Tyrosine, Propanoate and Phenylalanine metabolism, etc. In addition, MR analysis demonstrated a causal relationship between pyruvate metabolism and BC risk. Finally, we constructed a regulatory network between pathway genes (ADH1B, ACSS2, ACACB, ADH1A, ALDH2, and ADH1C) and targeted drugs, as well as a ceRNA (lncRNA-miRNA-mRNA) regulatory network for BC, further revealing their interactions.

**Conclusions:**

Our research revealed a causal association between pyruvate metabolism and BC risk, found that ADH1B, ACSS2, ACACB, ADH1A, ALDH2, and ADH1C takes place an important part in the development of BC in the molecular mechanisms related to pyruvate metabolism, and identified some potential targeted small molecule drugs.

**Supplementary Information:**

The online version contains supplementary material available at 10.1007/s12672-024-01125-7.

## Introduction

Breast cancer (BC) is a condition due to the unchecked growth of mammary epithelial cells as a result of several carcinogenic factors [1]. Nipple discharge, Breast lumps, enlargement of axillary lymph node and other symptoms are common in the early stages of the disease, and multiple organ lesions and distant metastases of cancer cells can cause multiple organ lesions in the late stages, which can directly jeopardize patients' lives [2, 3]. Although the precise etiology of BC is still unknown, scientists have discovered numerous risk factors that are linked to the disease's occurrence. Estrone and estradiol are associated directly to the occurrence of BC. Additionally, an increased risk factors for BC is genetic factors and some gene mutations can also raised the BC risks. Certain lifestyle choices, such as overindulging in food, becoming obese, maintaining a high-fat diet, binge drinking, etc., are related with a higher chance of developing breast cancer [4–7].

Pyruvate is a product of the catabolism of sugars and most amino acids. It can convert sugars, fats and amino acids into each other via the cycles of tricarboxylic acid and acetyl CoA [8]. As a result, it is crucial to the metabolic connections between the three main nutrients. High plasma pyruvate can be seen in vitamin B1 deficiency, diabetes, congestive heart failure, severe diarrhea and other digestive disorders, serious infection and liver disease can also be increased pyruvate, accompanied by hyperlactemia. Investigation has revealed that the end byproduct of the anaerobic glycolysis cycle is pyruvate, and large number of cancer cells use pyruvate for growth and development [9]. Abnormal pyruvate metabolism is associated with many human metabolic disorders and leads to diabetes, cancer, and heart disease [10]. Impaired function of mitochondrial pyruvate carriers may induce tumors with strong proliferation, migration and invasion capabilities [11]. Coincidentally, several researches have also found that pyruvate metabolism affects the proliferation, differentiation and metastasis of BC cell lines [12–14]. Reports on the link between pyruvate metabolism and BC risk, however, are not readily available. Some previous studies have shown that Mendel randomization can be used to predict and study breast cancer. For example, in a 2021 study, researchers assessed the potential causal impact of smoking on the risk of breast cancer through Mendel randomization analysis. The individual level data and summary statistical data of a total of 164 SNPs reported in lifetime smoking index or genome-wide association studies of daily cigarettes were used for MR evaluation. The researchers measured 2929 unique proteins in the plasma of 598 women selected from the Karolinska mammography project to explore the relationship between protein levels, clinical characteristics and gene variation, and identify the proteins that have causal relationships with breast cancer. They proposed 812 cis acting protein quantitative trait loci of 737 proteins, which were used as a tool for Mendelian random analysis of breast cancer risk.

In this study, we collected and analyzed various datasets from the GEO database to identify differentially acting genes (DAGs) in breast cancer (BC) through differential expression analysis. Subsequently, we conducted functional and pathway enrichment analyses to determine significant molecular functions and metabolic pathways in BC. Utilizing Mendelian randomization analysis, we established a causal relationship between pyruvate metabolism and the risk of BC. Furthermore, leveraging the DGIdb database, we identified potential targeted drugs that target genes involved in the pyruvate metabolic pathway and established a competing endogenous RNA (ceRNA) regulatory complex in BC.

## Materials and methods

### Data source and collation

The GENE EXPRESSION OMNIBUS (GEO) database was found in 2000 by the National Center for Biotechnology Information (NCBI). It functions as a comprehensive repository for gene expression data provided by research institutions worldwide, encompassing various types of data such as gene chips, high-throughput sequencing data, and other pertinent information [15]. In this study, we utilized datasets GSE54002, GSE70947, and GSE22820, which are Breast cancer Database, from the GEO database to procure three transcriptome gene expression matrices. The probe ids were converted into gene symbols. Subsequently, the samples were segregated as two different groups, such as the BC group and the Normal group, according to the clinical data file. Lastly, the R software packages limma and sva were employed to amalgamate these three gene expression matrices, resulting in the creation of a corrected gene expression matrix [16].

### Principal component analysis (PCA) of different datasets

In order to mitigate the presence of batch effects across distinct GEO datasets, we conducted batch correction on the amalgamated gene expression matrix. Subsequently, the efficacy of this correction was assessed through Principal component analysis (PCA) [17]. PCA is a method of statistics that employs orthogonal transformation to change possibly correlated variables sets into a linearly independent variables sets. These modified variables are referred to as major components [18]. To execute the PCA analysis on the expression matrix pre and post correction, we employed the R packages ggplot2 and ggpubr, utilizing the bioPCA function. The outcomes were then visually represented using scatter plots [19].

### Differentially expressed gene (DEG) analysis in BC

In order to ascertain differentially acting genes (DAGs) among BC and normal groups, a DEG analysis was conducted. This analysis entailed the utilization of the R program to input the compiled transcriptome gene expression matrix. By employing the wilcox.test function, a comparison of gene expression levels was performed between the two groups, resulting in the generation of an expression matrix and difference result parameters for the DAGs by the R package limma. Subsequently, the DAGs were visually represented through a heatmap and a volcano map [20, 21]. Statistical significance was found out by an adjusted P-value < 0.05 and an absolute log fold change (|lgFC|) of ≥ 0.585.

### Function and pathway enrichment analysis of DAGs

By employing Gene Ontology (GO) and Kyoto Encyclopedia of Genes and Genomes (KEGG) enrichment analyses were obtained a more comprehensive comprehension of the biological processes, molecular functions, and metabolic pathways of DEGs [22]. Initially, the R packages clusterProfiler and org.Hs.eg.db were utilized to conduct GO enrichment analysis of DEGs, encompassing the examination of biological processes (BP), cellular components (CC), and molecular functions (MF). Subsequently, KEGG enrichment analysis was conducted using the enrichKEGG function to identify significant biological pathways based on DEGs. Finally, the outcomes of the enrichment analyses were visually represented through bubble diagrams [23]. Significance was found out by adjusted P-value of < 0.05.

### Mendelian randomization analysis

A two-sample Mendelian randomization (MR) approach was utilized to investigate the causal association among biological pathways and the risk of BC. Single nucleotide polymorphism (SNP) was defined as the instrumental variable (IV). Exposure and outcome data were obtained from a publicly available Genome-Wide Association Study (GWAS) database (https://gwas.mrcieu.ac.uk/) [24]. Subsequently, the significant pathway Pyruvate metabolism (ID: ebi-a-GCST90092942) and the disease BC (ID: ebi-a-GCST007236) were chosen for the MR analysis. The MR analysis was conducted using the "TwoSampleMR" package, with the inverse variance weighted (IVW) method employed to examine the association among the activity level of Pyruvate metabolism and the risk of BC. We specified a threshold of P < 1 × 10^−5^ to select SNP instruments and LD r^2^< 0.1 threshold for clumping analysis to get independent genetic variants for MR analysis. Then, an unweighted polygenic risk score (PRS) was calculated for each individual using independent genetic variants from GWAS data. Each SNP was recoded as 0, 1 and 2, depending on the number of trait-specific risk increasing alleles carried by an individual. We performed Instrumental variable (IV) analyses employing two-stage least square regression (TSLS) method. In the first stage, for each exposure trait, association between the GRS and observational phenotype value was assessed using linear regression and predicted fitted values based on the instrument were obtained. In the second stage, linear regression was performed with outcome trait and genetically predicted exposure level from the first stage. In both stages, analyses were adjusted for age, gender and top four principal components of population structure. For each trait, TSLS was performed using ‘ivreg’ command from the AER package in R. Additionally, a sensitivity analysis was performed using MR-Egger [25].

### Analysis of the binding between pathway genes and targeted drugs

The Drug-Gene Interaction database (DGIdb) (https://dgidb.genome.wustl.edu/), serving as a comprehensive repository of Drug-Gene Interactions, offers genetic information pertaining to established and potential drugs [26]. In this investigation, the search field was initially populated with pathway genes, followed by the application of filtering conditions to specify the drug type as Approved, Antineoplastic and Immunotherapies. The default selections for database source, gene category, and interaction type were retained. Subsequently, the resulting gene-drug interaction file was downloaded. Ultimately, the software Cytoscape was employed to visualize the interaction between the pathway genes and the targeted drugs [27].

### Establishment of a ceRNA (lncRNA–miRNA–mRNA) regulatory complex

Initially, we employed miRanda, miRDB, miRWalk, and TargetScan databases to forecast the miRNAs that are bound by pathway genes. Subsequently, if all four databases concur on a specific miRNA as a potential target, it is retained as the designated targeted miRNA [28]. Additionally, we utilized the spongeScan database to anticipate the lncRNAs that target the binding of miRNAs and assigned names to the identified targeted lncRNAs [29]. Lastly, to depict the regulatory network involving circRNA-miRNA-mRNA, we employed Cytoscape software for visualization purposes.

## Results

### Gene expression matrix of BC

Initially, we compiled the datasets GSE54002 (consisting of 417 BC samples and 16 Normal samples), GSE70947 (comprising of 148 BC samples and 148 Normal samples), and GSE22820 (comprising of 176 BC samples and 10 NC samples). Subsequently, we acquired expression matrices containing 21,654, 27,440, and 19,593 genes, respectively (refer to supplementary files Matrix_GSE54002.xls, Matrix_GSE70947.xls, and Matrix_GSE22820.xls). Following this, we merged the three matrices to generate a refined expression matrix comprising 15,895 genes (comprising 741 BC samples and 174 Normal samples) (refer to supplemental file Matrix_merge.xls).

### PCA analysis of three datasets

**Fig. 1:**
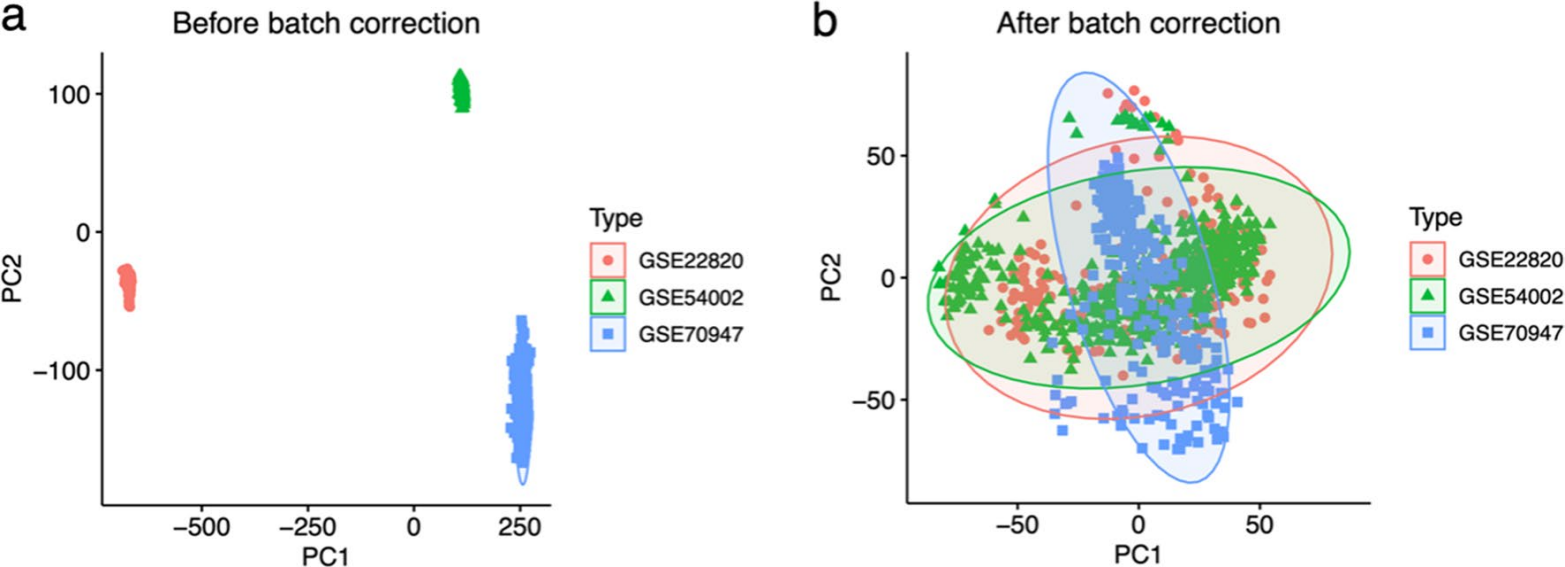
Scatter plots of principal component analysis before (**a**) and after (**b**) batch correction. PC1, principal component 1; PC2, principal component 2. Different colored dots indicate samples from different datasets

PCA was employed to visually depict the presence of batch effects across distinct datasets both prior to and subsequent to batch correction. The obtained results indicated a notable batch effect in the samples originating from three separate datasets prior to any batch correction (Fig. 1a). Conversely, following the application of batch correction, no significant batch effect was observed within the samples derived from the aforementioned three datasets (Fig. 1b).

### Identification of DEGs between BC and Normal groups

**Fig. 2:**
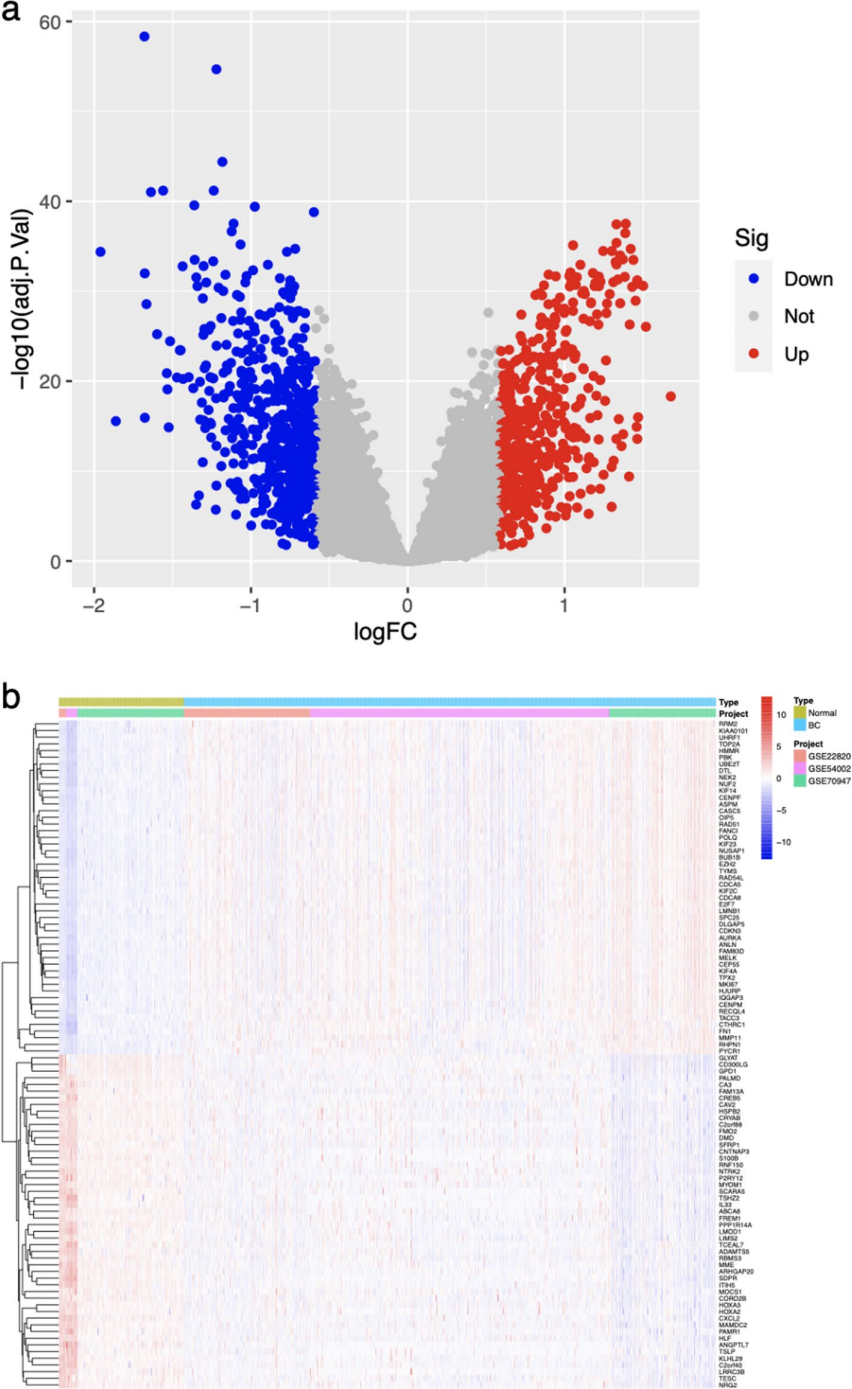
The volcano plot (**a**) and heatmap (**b**) of DAGs expressed between the BC and Normal groups. Red dots or squares indicate up controlled DAGs; Blue dots or squares indicate down controlled DAGs

By utilizing differential expression analysis, overall 1127 DEGs were recognized among the BC and NC groups. Among these DEGs, 523 showed up-regulation, while 604 exhibited down-regulation, as visually depicted in Fig. 2a. Furthermore, Fig. 2b presented a heat map illustrating the top 50 DEGs that displayed both up-regulation and down-regulation.

### GO and KEGG enrichment of 1127 DAGs in BC

**Fig. 3:**
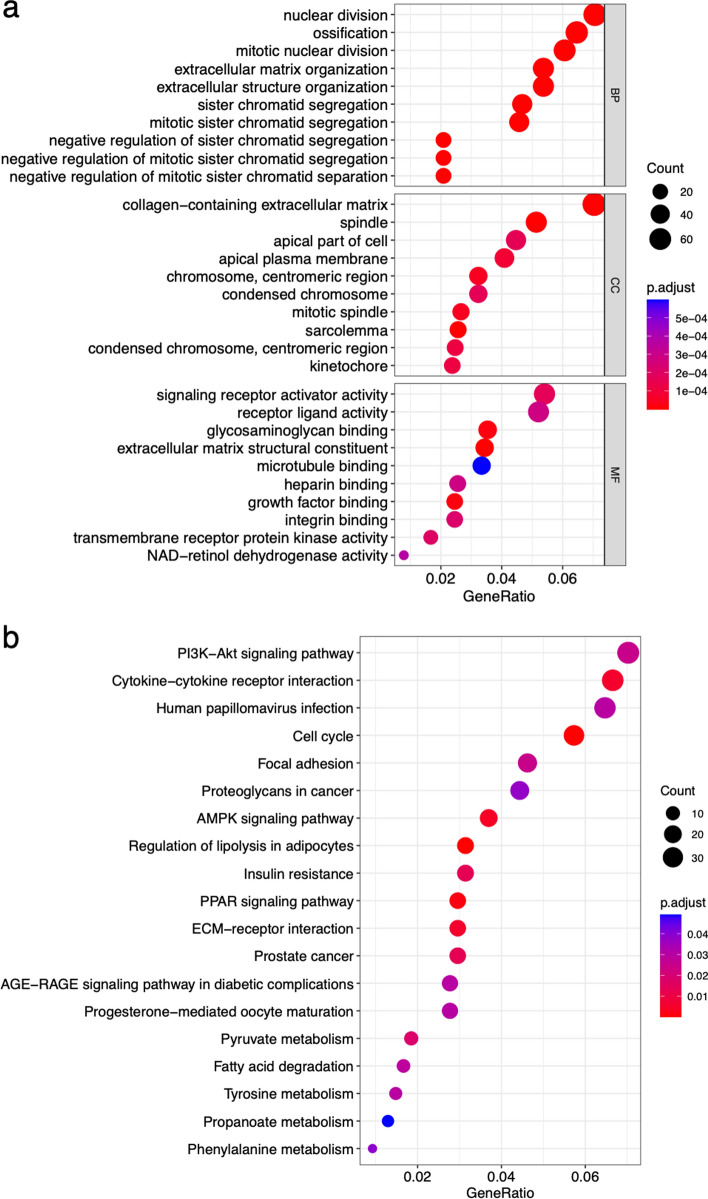
Bubble plots for GO (**a**) and KEGG (**b**) enrichment analyses. Different GO and KEGG terms are displayed on the ordinate, and gene enriched ratiosaredisplayed on the abscissa

Through the examination of GO and KEGG enrichment, we have identified the biological processes, molecular functions, and metabolic pathways of DAGs. As depicted in Fig. 3a, the GO enrichment analysis revealed that the functions of BP primarily encompass nuclear division, ossification, mitotic nuclear division, organization of extracellular matrix and segregation of sister chromatid. The functions of CC were found to be closely linked to collagen-containing extracellular matrix, mitotic spindle, apical plasma membrane, and centromeric region. The functions of MF were predominantly enriched in signaling receptor activator activity, glycosaminoglycan, microtubule, heparin, growth factor and integrin binding, extracellular matrix structural constituent. In the KEGG enrichment analysis, the pathways identified by DEGs were primarily focused on PI3K−Akt signaling pathway, Cytokine−cytokine receptor binding, Human papillomavirus infection, AMPK signaling pathway, Regulation of lipolysis in adipocytes, Insulin resistance, PPAR signaling pathway, ECM-receptor interaction, Fatty acid degradation, Pyruvate, Tyrosine, Propanoate and Phenylalanine metabolism, among others (Fig. 3b).

### Pyruvate metabolism was causally correlated with the BC risks

**Fig. 4:**
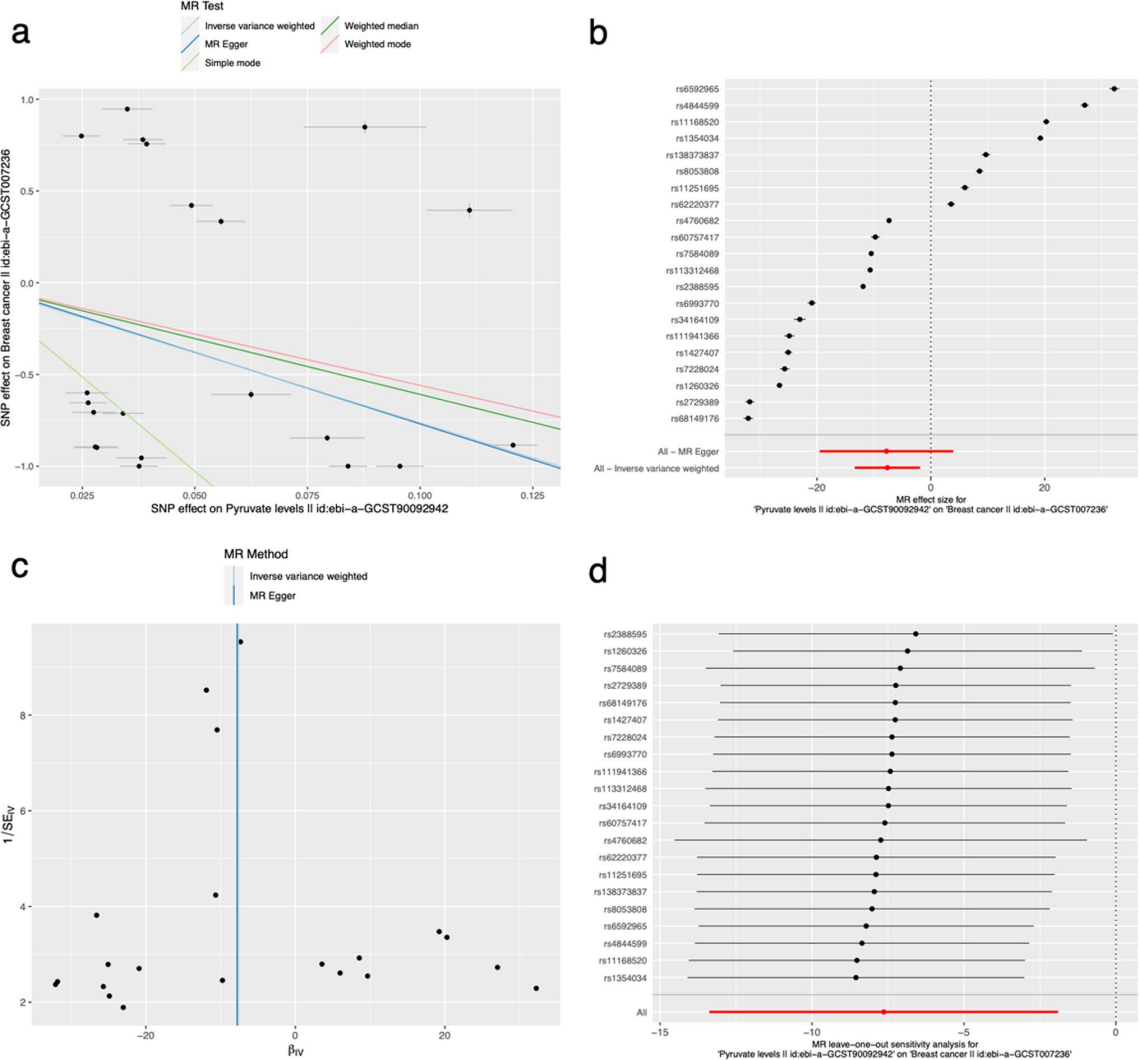
**a** Scatter plot illustrating the causal effect of Pyruvate metabolism on the risk of BC. **b** Forest plot displaying the causal effect of each SNP on BC risk. **c** Funnel plots used to visualize overall heterogeneity of MR estimates for the effect of Pyruvate metabolism on BC. **d** Leave-one-out plot allowing for the visualization of the causal effect of Pyruvate metabolism on BC risk when leaving one SNP out

The Supplementary file SNP.xls provided an overview of the SNP characteristics related to Pyruvate metabolism and BC. None of the SNPs were deemed weak instrumental variables. Figure 4a and b depicted the causal genetic difference effect on BC. By employing the IVW method, were observed a remarkable correlation between the activity level of Pyruvate metabolism and the risk of BC, with an odds ratio (OR) of 0.00048 (95% confidence interval (CI) = 1.556e−06 to 0.150, p = 0.009). Additionally, Simple mode, the Weighted median and Weighted mode methods also yielded statistically significant findings (see Table 1).The symmetrical characteristics exhibited by the funnel plot indicate the presence of a causal effect (Fig. 4c). Furthermore, the absence of horizontal pleiotropy in the MR Egger regression (p = 0.975) provides additional evidence of the lack of bias in the causal effect. After removing each individual SNP, we ran MR analysis on the remaining SNPs to confirm the validity of our results, as shown in Fig. 4d. The consistent outcomes obtained from this analysis suggest that all SNPs make significant contributions to the observed causality. This implies that there is no singular dominant SNP exerting influence on Pyruvate metabolism and the risk of BC.

**Table 1:** Statistical results of different Mendelian randomization methods

Method	Nsnp	B	SE	P-value	Lo_ci	Up_ci	OR	OR_lci95	OR_uci95
MR Egger	21	− 7.8002	5.9906	0.2085	− 19.5418	3.9414	0.0004	0.0000	51.4924
Weighted median	21	− 6.0890	0.3658	0.0000	− 6.8060	− 5.3721	0.0023	0.0011	0.0046
Inverse variance weighted	21	− 7.6365	2.9271	0.0091	− 13.3736	− 1.8994	0.0005	0.0000	0.1497
Simple mode	21	− 20.5532	2.1320	0.0000	− 24.7320	− 16.3744	0.0000	0.0000	0.0000
Weighted mode	21	− 5.5964	0.3427	0.0000	− 6.2680	− 4.9247	0.0037	0.0019	0.0073

### Regulatory complex of pathway genes and targeted drugs

**Fig. 5:**
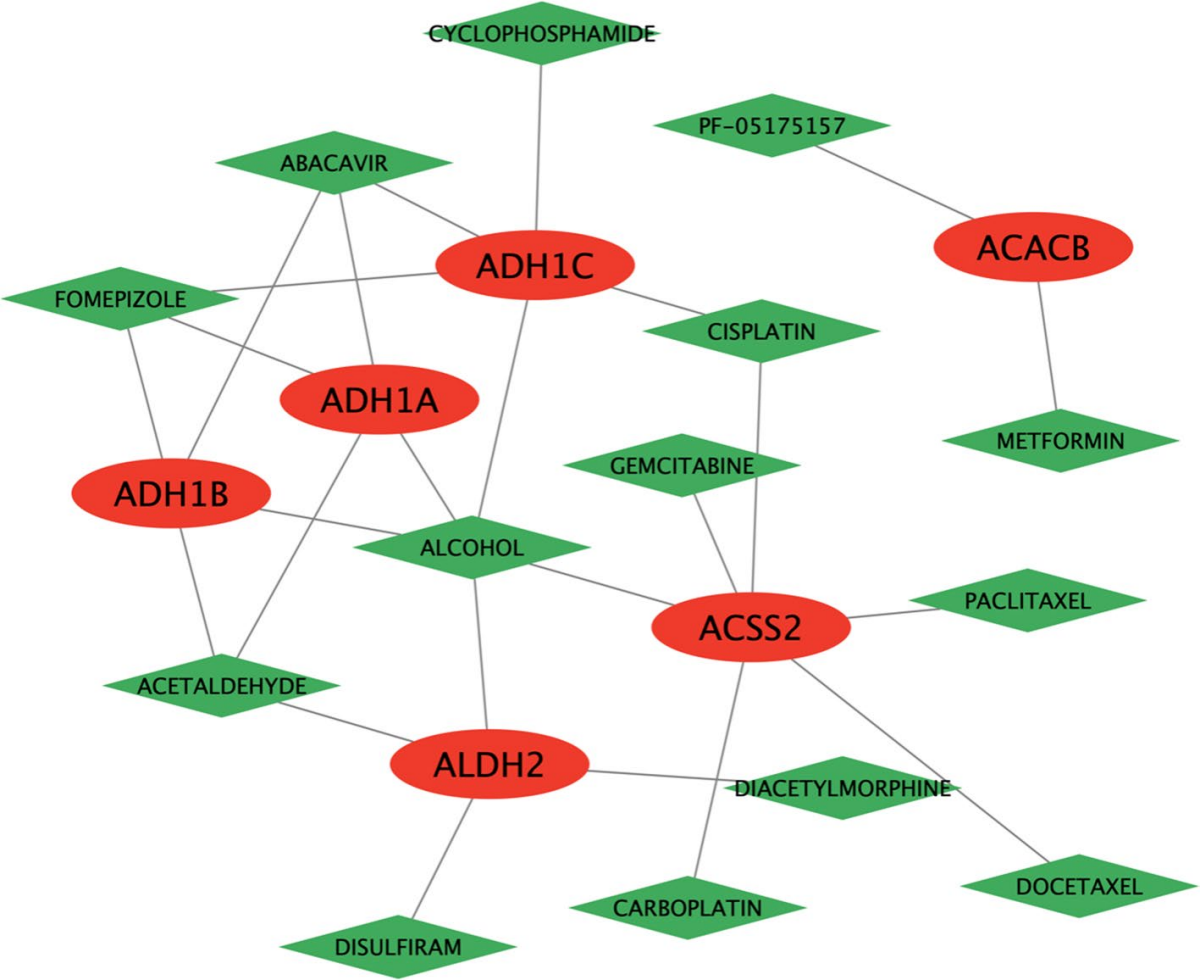
Regulatory complex between pathway genes and targeted drugs. The red nodes represent pathway genes, the green nodes represent targeted drugs, and the lines between them represent interaction pairs

The DGIdb database was utilized to acquire a comprehensive table encompassing pathway genes and their corresponding targeted drug interactions, as described in the supplementary file (Drug-gene.xls). Subsequently, a regulatory complex was established by Cytoscape to visually represent the connections between pathway genes and targeted drugs. The graphical representation in Fig. 5 depicted pathway genes as red oval nodes and targeted drugs as green diamond nodes, with the lines connecting these nodes representing their interactions. This drug regulatory network encompassed a total of 6 pathway genes (ADH1B, ACSS2, ACACB, ADH1A, ALDH2, and ADH1C), 14 molecular drugs, and 25 pairs of interacting relationships.

### The ceRNA (lncRNA–miRNA–mRNA) regulatory complex of BC

**Fig. 6:**
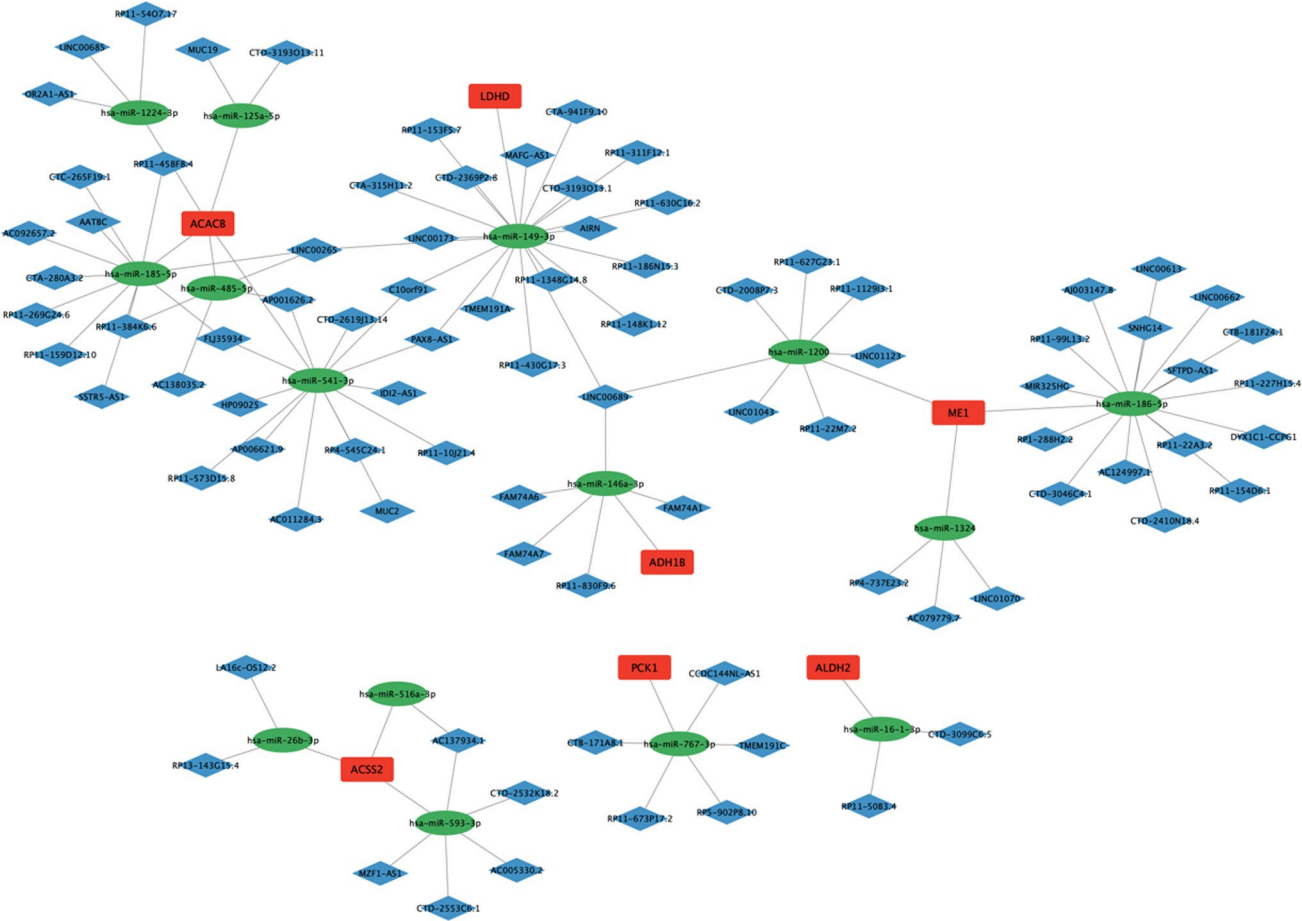
The ceRNA (lncRNA-miRNA-mRNA) regulatory complex of BC. Red square nodes indicate mRNAs, green elliptic nodes indicate miRNAs, and blue diamond nodes indicate lncRNAs. The lines between them represent interactions between different types of RNAs

Overall 47 pairs of mRNAs and miRNAs, as well as 98 pairs of lncRNAs and miRNAs, were acquired from various databases (refer to Supplementary filesmRNA-miRNA.xls and miRNA-lncRNA.xls). The ceRNA (lncRNA-miRNA-mRNA) regulatory complex (Fig. 6) was employed to visualize the interactions among these entities. This regulatory network comprised a total of 7 mRNAs, 15 miRNAs, 88 lncRNAs, and 113 pairs of interacting relationships.

## Discussion

One of the most prevalent cancerous tumors in women is BC. Based on statistical data, 7–10% of all malignant tumors in the body are accounted for by this incidence rate. An occurrence is often associated with heredity and the rate of occurrence is greater in women between 40 and 60 years old and before and after menopause [30, 31]. Pyruvate is a tricarboxylic acid produced in the body, which is the end product of the glycolytic pathway. For energy, it is either oxidized into acetyl CoA in mitochondria or reduced to lactate in the cytoplasm. After entering the tricarboxylic acid cycle, it undergoes oxidation to produce carbon dioxide and water, consummating the energy supply process of aerobic oxidation of glucose [32]. Through the acetyl CoA and tricarboxylic acid cycles, pyruvate can also accomplish mutual conversion between sugars, lipids, and amino acids in the body, and it plays a crucial part in the metabolic relationships of the three main nutrients [33]. This article identified a total of 1127 DEGs in the BC and NC groups through differential expression analysis. Subsequently, the biological processes, molecular functions, and metabolic pathways of DEGs were determined through GO and KEGG enrichment analysis. Furthermore, through Mendelian randomization analysis, it was demonstrated that there is a causal relationship between pyruvate metabolism and the risk of BC. Finally, we constructed a regulatory network between pathway genes and targeted drugs, as well as a ceRNA (lncRNA miRNA mRNA) regulatory network for BC, further revealing their interactions.

Our study not only revealed a causal relationship between pyruvate metabolism and BC risk, but also found that ADH1B, ACSS2, ACACB, ADH1A, ALDH2, and ADH1C takes place a major part in the development of BC in the molecular mechanisms related to pyruvate metabolism, and identified some potential targeted small molecule drugs. Our comprehensive analysis links GWAS and transcriptomics together.

In our study, we indirectly demonstrated a causal association among pyruvate metabolism and the BC risks. As the activity of the pyruvate metabolism pathway increases, the risk of developing BC decreases (OR < 1 and B < 0). Therefore, the pyruvate metabolism pathway may have a protective causal relationship in BC. By comparing ^13^C tracer analyses, Christen S et al. [14] found that lung metastatic stoves had higher pyruvate carboxylase (PC)-dependent repair to adapt to the lung microenvironment compared to primary BC. The Elia I team [13] found that in order to stimulate collagen-based remodeling of the extracellular matrix in the lung metastatic niche, BC cells need the nutrient pyruvate. Nonetheless, in many animal models, it was sufficient to restrict pyruvate metabolism in order to hinder collagen hydroxylation and, consequently, the formation of lung metastases originating from BC.

The gene ADH1A (Alcohol dehydrogenase 1A (class I), alpha polypeptide), ADH1C (Alcohol dehydrogenase 1C (class I), gamma polypeptide) and ADH1B (Alcohol dehydrogenase 1B (class I), beta polypeptide) encode proteins that belong to the alcohol dehydrogenase family, which are known for its capacity to metabolize a diverse range of substrates such as retinol, ethanol, hydroxysteroids, aliphatic alcohols and lipid peroxidation products. These proteins, composed of various homo- and heterodimers of alpha, beta and gamma subunits, demonstrate significant effect in ethanol oxidation and are crucial in the process of ethanol catabolism [34–36]. Jiang C et al. [37] determined that ADH1B may suppress the invasion, proliferation and migration of BC cells by suppressing mitogen-activated protein kinase (MAPK) signaling pathway, so it may be a potential clinical therapeutic target for BC. Coutelle C et al. [38] found a significant increase in the allele frequency of ADH1C*1 in the moderate drinking group with BC compared to the normal control group who drank alcohol (62% vs. 41.9%, p = 0.0035). Women with the ADH1C*1 genotype had a 1.8 times greater risk of developing BC than women with other genotypes (95% CI 1.431–2.330, p < 0.001).

This geneACSS2 (Acyl-CoA synthetase short chain family member 2) encodes a cytosolic enzyme responsible for catalyzing the acetate activation to be utilized in synthesis of lipids and production of energy. This enzyme functions as a monomer and facilitates the conversion of acetate to acetyl-CoA in an ATP-dependent reaction [39, 40]. Miller KD et al. [41] synthesized a small molecule inhibitor as a transition state mimetic to block ACSS2 activity in vitro and in vivo, while ACSS2 acts as an individual drug to inhibit BC cell growth. This indicates that targeting ACSS2 may be an effective method for treating BC patients.This geneACACB (Acetyl-CoA carboxylase beta) is believed to regulate fatty acid oxidation through the inhibitory effect of malonyl-CoA on carnitine-palmitoyl-CoA transferase I, the crucial step in mitochondrial fatty acid uptake and oxidation [42]. Yang JH et al. [43] observed that combined inhibition of the pentose phosphate pathway and fatty acid oxidation (FAO) with clinically available drugs effectively restored SNAI1, a basic transcriptional suppressor of epithelial-mesenchymal transformation, mediates reprogramming of metabolism and inhibits the in vivo metastasis progression in BC cells. This protein encoded by gene ALDH2(Aldehyde dehydrogenase 2 family member) is classified within the aldehyde dehydrogenase protein family, which plays a key role as the second enzyme in the primary oxidative pathway of alcohol metabolism [44].Ugai T et al. [45] found that functional ALDH2 polymorphisms are correlated to the BC risks, and Lys/Lys genotypes confer susceptibility to BC risk in Asian women, especially for ER-positive, PR-positive, and HER2-negative tumor types. These previous studies are similar to our results.

The main advantage of this study is that it fully incorporates various metabolic processes and strict MR design. The data related to BC comes from the GEO database to ensure the richness and credibility of the data. The causal relationship between the pyruvate metabolism pathways of BC was determined through MR analysis, and further confirmed through meta-analysis and MVMR methods. Conduct in-depth research on causal relationships using mediation MR analysis. Therefore, we believe that our research findings are convincing and provide reliable causal explanations for the impact. These findings may provide valuable insights for prospective treatment of BC by further investigating the mechanisms associated with BC. However, our investigation has some limitations. Firstly, we cannot determine the applicability of our population analysis based on this study to other populations. Although our research findings have demonstrated a causal relationship between the pyruvate metabolism pathways of BC and identified potential mediators, further research is needed to explore their potential mechanisms in order to develop effective and feasible treatment methods for BC due to data limitations. Besides, our study is limited by the content and workload of the study, and further verification on protein levels and large sample clinical trials is needed in the future. In addition, we ultimately did not use BC's pyruvate metabolism pathway genes and potential drugs in animal studies to verify its effects. These will be the subjects of our upcoming studies.

The pyruvate metabolism pathway of BC is an important health issue today, although there seems to be a causal relationship between them, it is currently unclear. This study was the first to use the MR method to test this relationship. By using instrumental variables, we evaluated the causal relationship between the pyruvate metabolism pathways of BC. In addition, mediating MR helps us understand the role of the pyruvate metabolism pathway in this relationship. These findings lay the foundation for promoting the understanding and treatment of BC, with the ultimate goal of reducing incidence rate, mortality and the burden of the global health system.

## Conclusions

Our study demonstrated a causal association among pyruvate metabolism and the risk of BC, and found that ADH1B, ACSS2, ACACB, ADH1A, ALDH2, and ADH1C play a major part in the molecular mechanisms related to pyruvate metabolism in the occurrence of BC, and identified some potential targeted small molecule drugs. In addition, the regulatory network of ceRNA (lncRNA-miRNA-mRNA) further revealed the interactions between mRNAs related to this pathway.

### Supplementary Information

Additional file 1.

Additional file 2.

## Data Availability

The data are available at the GEO database (https://www.ncbi.nlm.nih.gov/geo/) (GSE42568), TCGA database (http://cancergenome.nih.gov/), and TCIA database (https://tcia.at/).
